# What Are the Roles and Responsibilities of the Media in Disseminating Health Information?

**DOI:** 10.1371/journal.pmed.0020215

**Published:** 2005-07-26

**Authors:** Gary Schwitzer, Ganapati Mudur, David Henry, Amanda Wilson, Merrill Goozner, Maria Simbra, Melissa Sweet, Katherine A Baverstock

## Abstract

Background to the debate: In December 2004 three news stories in the popular press suggested that the side effects of single-dose nevirapine, which has been proven to prevent mother-to-child transmission of HIV, had been covered up. Many HIV experts believed that the stories were unwarranted and that they would undermine use of the drug, leading to a rise in neonatal HIV infection. The controversy surrounding these stories prompted the *PLoS Medicine* editors to ask health journalists, and others with an interest in media reporting of health, to share their views on the roles and responsibilities of the media in disseminating health information.

## Abstract 2

The way in which the media report health issues has recently come under scrutiny. What are the the duties of health reporters to their readers?

## Gary Schwitzer: The Agenda-Setting Role of Health Journalists

Some journalists say that their role and responsibility is no different in covering health information than it is in covering politics, business, or any other topic. These journalists say that their primary concern is accurate, clear reporting—they are less concerned about the consequences of their story once it is published [1]. But that approach may result in shoddy journalism and potential harm to the public [2]. I assert that it isn't sufficient to be accurate and clear when covering health news. Journalists have a responsibility to mirror a society's needs and issues, comprehensively and proportionally [3]. Often that doesn't happen in health news.

Recently, I led an effort by the Association of Health Care Journalists to publish a statement of principles [4]. “Journalists have a special responsibility in covering health and medical news,” the statement reads. “Association members know that readers and viewers may make important health care decisions based on the information provided in our stories.”

In our current era of entanglement, journalists must investigate and report the possible conflicts of interest among sources of health information and those who promote a new idea or therapy. Such conflicts may not be readily apparent, so journalists must look for them as a routine part of story research and interviews. They must investigate and report the possible links between researchers and private companies, researchers and public institutions, patient advocacy groups and their sponsors, celebrity spokespersons and their sponsors, and nonprofit health and professional organizations and their sponsors. To fail to do so may mean that journalists become unwitting mouthpieces for incomplete, biased, and imbalanced news and information.

Journalists face unique challenges in covering health news. Some specialized skills, knowledge, and judgment are helpful. For example, some information based on poorly designed or poorly powered studies should not be reported unless the flaws are emphasized.

Editors, reporters, and writers need to scrutinize the terminology used in health news. Vague, sensational terms (such as “cure,” “miracle,” and “breakthrough”) may harm news consumers by misleading and misinforming [5]. At the core of journalism's values, such terms should not be used because they are meaningless.

It is not the role of journalists to become advocates for causes. However, I believe that journalists have a responsibility to investigate and report on citizens' needs as they struggle to understand and navigate the health-care system. People need help in understanding the ways in which scientists and policymakers reach conclusions. In that sense, there is an inherent educational role that journalists must assume. [Fig pmed-0020215-g001]

**Figure pmed-0020215-g001:**
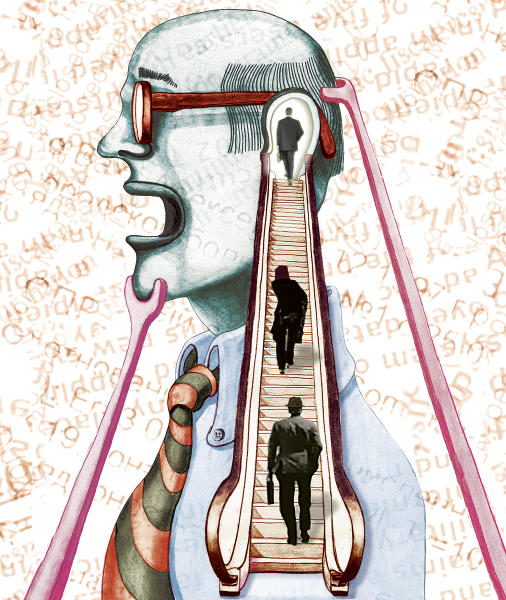
Journalists risk becoming unwitting mouthpieces for those with vested interests in their story (Illustration: Scott Mickelson)

I have a special interest in how television journalists cover health and health policy news. Surveys consistently show that many Americans get most of their health news and information from television. One study documented troubling trends of brevity (an average of 45 seconds per story), absence of reporter specialization, sensational claims not supported by data, hyperbole, commercialism, disregard for the uncertainty of clinical trials, baseless predictions of treatments based on basic science studies, single-source stories, and a paucity of coverage of health policy [6].

Television viewers are likely to see many more one-sided political ads about health policy issues than balanced, comprehensive news stories about such issues. In my current research, I am analyzing health policy news coverage on three award-winning TV stations in three different parts of the United States in 2004. Despite the fact that American voters ranked health care as their third leading concern (after war and the economy) [7], the three stations I monitored devoted little time to health policy issues. My analysis shows that in ten months (326 hours of stations' key late night newscasts) on these three stations, there was only one story on the uninsured. Presidential candidates' health policy platforms drew a combined total of seven minutes of news—an average of 23 seconds per story, or about 15 seconds per station per month of the 2004 campaign. Whether it is preclinical news that is not ready for prime time, or clinical news that oozes optimism over unproven ideas, or a disdain for health policy news, television journalists seem to have abdicated their possible agenda-setting role.

Journalists must weigh the balance between the amount of attention given news about medicine and the attention given news about health and the social determinants of health. There may be too much news about the delivery of medical services and not enough news about the cost of, quality of, and evidence for those services. The current imbalance may contribute to the nation's health-care cost crisis, driving up demand for expensive, unproven ideas. These are responsibilities journalists may not encounter in covering other topics. In health news, they are everyday issues.

## Ganapati Mudur: The Media May Be the Most Important Source of Health Information for the General Public

Health reporting does involve “telling a story,” but it also requires writers to take on additional responsibilities through the story cycle—finding the story, collecting information, and writing it.

Standard news criteria such as timeliness and impact may be used to pick stories. But in health reporting, context is crucial. Research advances to be reported need to be placed in context. This may be achieved by citing earlier research on the topic and seeking out comments from independent experts who could put a new finding in perspective. Sometimes health research throws up contradictory findings. Is a gene linked to a disease? One study finds a link. Another does not. Such situations demand interpretative and analytical skills on the part of health writers. Otherwise, writers may mislead readers, or leave them confused.

Health reporters need to find out who has funded the research and who might be likely to gain. And reporters must always double-check claims or else they may end up in embarrassing situations. Let me illustrate with an example. A top international science magazine last year reported that a novel stem cell therapy had cured patients with chronic aplastic anemia in Bombay, India [8]. The story was apparently based on claims made by the developers of the therapy, a private British company. A little more patience and investigation would have led the magazine to the real story: none of the patients had responded to the treatment, and the clinical collaborators in India had terminated the study [9].

When a public health situation is involved, health writers and the media can certainly play a role in quickly delivering important messages to the public. In a sense, then, they do serve as a component of the health provider community. And this makes it all the more important for health writers to ensure that they get it absolutely right. Given that most people do not interact with their doctors on a regular basis, the media is possibly the most significant source of health information for the general public. But health information in the media cannot substitute for personal medical advice. It is important that the public understands this.

Regulatory mechanisms may be lax in some developing countries. India, for instance, has had a long history of unethical or illegal clinical trials. Drug regulatory authorities in India allow the sale of drugs—including pediatric formulations—that have never been approved in Western countries. This opens up opportunities for investigative health journalism, an opportunity for reporters to take up the traditional watchdog role of the press to find and report wrongdoing.

## David Henry and Amanda Wilson: Health Journalists Should Discuss Benefits and Harms of New Treatments and Use Independent Expert Sources

Health reporting is a major growth area for the media, probably because it is in demand by the public and it is profitable. However, media coverage of medical news is generally of poor quality, particularly stories about new treatments [10–12].

Media Doctor is a Web site where the quality of stories in the Australian press is reviewed (www.mediadoctor.org.au). We rate articles using ten evaluation criteria (see Box 1; www.mediadoctor.org.au/content/ratinginformation.jsp). In February/March 2005 articles that we rated achieved an average of only 52% on our “satisfactory” score (www.mediadoctor.org.au/content/sourceinfo.jsp). This was an improvement on the score from one year ago, but it is still inadequate. North American analyses of the quality of health reporting have had similar results [11]. The print media are clearly superior to the online news services [12]. The greatest differences between print and online services are in the use of independent information sources, and the quantification of the benefits and the coverage of potential harms of new treatments.

**Box 1. Criteria Used by Media Doctor to Evaluate News Stories**

- Whether the treatment is genuinely new
- The availability of the treatment in Australia
- Whether alternative treatment options are mentioned
- If there is evidence of disease mongering in the story
- If there is objective evidence to support the treatment
- How the benefits of the treatment are framed (in relative or absolute terms)
- Whether harms of the treatment are mentioned in the story
- Whether costs of the treatment are mentioned in the story
- Whether sources of information and any known conflicts of interest of informants are disclosed in the article
- Whether the journalist relied only on the press release for the story

We recognise that there are different depths of journalism and that journalists face constraints, including commercial pressures and deadlines that give little time to reflect on stories, which are usually written on the same day as the press release arrives. Some journalists argue that the media are the messengers and not the message, and it is up to others to interpret their reporting. To a reporter who might otherwise exercise more caution, a well-written media release from a large public relations company describing a new pharmaceutical product must be attractive when a deadline is imminent. There is no danger that the company will allege plagiarism if it appears, almost intact, under the journalist's by-line.

And even when they do have the time, journalists face two major challenges—understanding the clinical science and epidemiology, and dealing with powerful vested interests. Vested interests are not unique to medicine, but reporting on a new drug is different from, say, an MP3 player or a dishwasher. People will be intensely interested in a story about a new drug if it purports to treat a condition that they or their relatives have, and the story may become the basis of discussions with their physician and subsequent treatment decisions. We believe that in writing this type of story journalists have special responsibilities to ensure that they provide balanced information for their readers. In Australia, the Press Council believes the matter is of sufficient importance to provide advice to journalists [13].

In our view journalists will meet their responsibilities if they cover certain key issues when writing stories about new medical treatments. These include the accurate reporting of the comparative benefits, harms, and costs of the treatment and the extent to which their informants have ties with the manufacturer. It is helpful if journalists use independent expert sources to answer questions about the novelty of the treatment and the availability and efficacy of alternatives, although we acknowledge the practical difficulties in finding independent sources when time is limited. Journalists have indicated to us that they are concerned about these issues and are prepared to look critically at their own practices. It is unclear whether their editors and producers hold the same views and will provide the necessary resources, particularly time to do the job properly.

But researchers and medical journal editors have responsibilities too. When reading medical news stories it is sometimes possible to tell whether the researchers and journals have done a good or bad job in communicating the essential facts to journalists. A number of medical journals issue press releases, and these have been found wanting [14]. Researchers should consider carefully what they wish to convey about the results of a new study and should ask to see and edit any press releases. We believe the criteria used by Media Doctor to evaluate news stories are a good starting point for researchers and editors.

## Merrill Goozner: Medical Reporters Must Get Beyond the Hype and Hope When Reporting on the Latest “Breakthrough”

When I broke into the news business, the financial desk's primary source of breaking news was a Dow Jones wire clack-clack-clacking in the corner of the room. A bell rang whenever a major story broke. Sometimes two bells would go off, signaling a really big story. The day the stock market crashed in 1987, the newsroom sounded like St. Peters Square on Easter.

I imagine something comparable occurs these days when the advance copies of leading medical journals cross science editors' computer screens. Stories from the frontiers of medical research can make it onto page one—the most coveted real estate in daily journalism. News magazines have bolstered their sagging bottom lines with an endless stream of cover stories touting the latest breakthroughs in medicine.

But is this news all that it is cracked up to be? Have the reporters properly weighed the importance of the studies they're touting? Have they asked the tough questions of the researchers and their sponsors to figure out the significance of the results? Have they presented the data in a fashion that is meaningful to health-care consumers? And in an age when most clinical trials are sponsored by private companies, have they fully informed their readers of the researchers' conflicts of interest?

Too often, the answer to these questions is no. Take recent reports from the American Society of Clinical Oncology, which met in mid-May in Orlando. One leading paper reported on a Veterans Administration review of the experience of over 40,000 women in the south central US. “The women taking statins were half as likely to have breast cancer as women who were not taking the drugs,” the paper reported [15]. Put that way, it sounds like a dramatic reduction. But elsewhere in the story, it was reported that 12 percent of the women were taking the cholesterol-lowering medications and that only 1.4 percent of the total group contracted breast cancer. Only by massaging the numbers could one figure out that physicians would need to put 700 women on statins to eliminate one cancer case (in medical parlance, this is called number needed to treat). It sounds a lot less impressive that way. But the number needed to treat would be a lot more meaningful to women, especially those on tight budgets wondering if it is worth $1,000 a year for a prescription.

Reporting of surrogate endpoints instead of primary endpoints is another way that readers get misled. Reports on cancer drug trials often fall into this trap. A “lifesaving” drug that shrinks tumors by 50 percent sounds a lot better than a chemotherapy agent that prolongs life by two months. The same can be said for bone density and fractures, blood pressure and strokes, and cholesterol levels and heart attacks. While there may be a minor yet statistically significant reduction in the primary endpoint, the trial sponsors prefer to promote the more dramatic-sounding secondary endpoint. Too many reporters prominently feature the less meaningful number, while leaving out or delaying until late in the story the real bottom line [16].

Sadly, the media have only lately come around to taking seriously the issue of conflicts of interest in medical science. Last July, the National Heart, Lung, and Blood Institute's National Cholesterol Education Project updated its guidelines for cholesterol management. The update, touted in the front page of every major US paper [17], called for a dramatic reduction in the cholesterol levels now considered optimal for people who have never had heart disease but are considered moderately at risk. Prescribing physicians using these guidelines will likely put millions more Americans on these drugs in the next few years.

Yet three days after the report came out, reporters at *Newsday* broke the story that eight of nine physicians on the National Cholesterol Education Project panel had financial ties to statin manufacturers, which had the most to gain from the new guidelines. Writing in the *Washington Post*, former *New England Journal of Medicine* editor Jerome Kassirer asked, “Why should we swallow what these studies say?” [18] The ensuing uproar contributed to a change in policy at the *New York Times*, which last fall circulated a memo to all reporters encouraging them to always report conflicts of interest of quoted sources in science stories, a policy that leading science and medical journals have had in place for many years [19].

In recent years the pharmaceutical and biotechnology industries have responded to complaints about the high cost of drugs by claiming they are needed to finance the medical miracles that are just around the corner. Meanwhile, the increase in life expectancy in the US has slowed and still remains far below other advanced industrial countries. The number of new drugs coming out of industry labs, despite a slight uptick last year, is actually down from a decade ago. In a health-care environment that is increasingly cost-constrained, it shouldn't be too much to ask that medical reporters get beyond the hype and hope when reporting on the latest “breakthrough.”

## Maria Simbra: Whatever News Managers Want, Viewers Get—As Medical Reporters Are Pressed to Feed the Media Beast

Reporters are surpassing doctors as a source of medical information. It's no secret health news sells. Producers and news directors take advantage of this to attract an audience for their newscasts. And viewers respond.

In a survey by Rodale Press, 39% of the respondents said they turn to TV for health and medical information, and 37% said they would ask a health professional [20].

So as audience-appointed proxies for “health professionals,” television medical reporters have a daunting task. They must be accurate, authoritative, and compassionate. They also need to understand the terminology, physiology, epidemiology, study design, and statistical analysis to keep health news in context for the viewer.

But typically, this doesn't happen. The medical industry churns out volumes of information for medical reporters to quickly sift through every day. There's a lack of special training for medical journalists (the general assignment reporter can expect to get thrown into the medical beat from time to time). Usually local news reports are under 90 seconds. The pressure for ratings compounds the problem.

Medical news is often simplified, or worse, sensationalized, because of industry pressures. Because health news sells, it can be and will be promoted—and in the process, distorted.

What is a medical reporter to do? Well, alone, there's not much a reporter can do. Like medical errors, the problems with medical journalism are system wide. At the root is a clash of cultures.

Medicine tends to be very methodical, slow, and subject to change. But the media want information that's definitive, they want it now, and, boy, it better be sensational. Also, people who go into journalism and ascend to management tend to be more inclined toward writing and creative interests. They may not understand (or may be openly hostile toward) the scientific process. [Fig pmed-0020215-g002]

**Figure pmed-0020215-g002:**
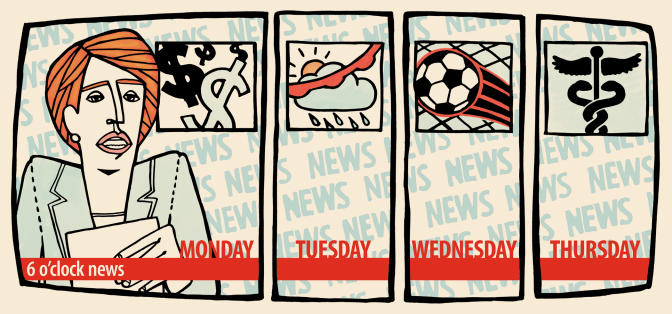
TV reporters rarely cover medicine exclusively—one day it's finance, the next it's health (Illustration: Giovanni Maki)

For TV reporters who are committed to the medical beat, educational opportunities are available through organizations such as the Association of Health Care Journalists (www.ahcj.umn.edu) and the National Association of Medical Communicators (www.ibiblio.org/namc). Journalists can learn how to interpret studies and present evidence-based balance in order to help viewers understand and make up their own minds about the latest developments in medicine, rather than just show the gee-whiz side of new technology.

Unfortunately, it's rare to find a TV reporter who exclusively covers medicine. Stations view this as a luxury. It is more common for medical reporters to also be general-assignment reporters or anchors, and they have other priorities with their combination jobs.

It's also rare to find management that's supportive of continuing education for their reporters. Even with the large profit margins at many TV stations, news directors generally do not provide financial support and ask that reporters interested in attending educational meetings use their own vacation time to do so. Furthermore, news directors, producers, and promotions staff don't seem to be themselves interested in learning about medical reporting.

An article in *JAMA* says that viewers are acting on and making personal medical decisions based on health information in the mass media [21]. This trend has led some TV medical reporting experts, such as Gary Schwitzer (formerly of CNN, now at the University of Minnesota, and a contributor to this *PLoS Medicine* debate) and Dr. Timothy Johnson *(ABC News)*, to call for credentialing of medical journalists. After all, some meteorologists are credentialed. Are personal health decisions less important than the weather?

There's a disconnect between what station management values, what the reporters need, and what the viewers get. Right or wrong, the audience looks to TV medical reporters to educate and guide them on medical issues. It's an important responsibility that medical reporters and the mass media in general need to take seriously.

## Melissa Sweet: Remember the Commercial Imperative When Examining Media Coverage of Health

Many people make the mistake of using the terms “journalists” and “the media” interchangeably. They speak, in the same breath, of the terrible failings of journalists and the media in covering health or other issues. In so doing, they fail to make a distinction between the craft or profession of journalism and the competitive industry that is the media. They fail to understand that the goals and drivers of journalism are often in conflict with those of the media industry.

The foremost goal of the media industry is, not surprisingly, to make profit. Many journalists are too idealistic to admit, even to themselves, that their job is to make money for their employers. Some believe they are there for the public interest, or even to interest the public. Some simply love to tell a yarn, to get the buzz that comes with uncovering a great story and breaking news. Some no doubt also come to enjoy the reflected glory of associating with the famous and the powerful. Indeed, many journalists have become celebrities themselves. Not coincidentally, this has benefits for their employers—nothing sells like celebrity.

But only a brave, naïve, or foolhardy journalist would publicly admit these days to believing that one of their roles is to help provide a voice for those who otherwise have difficulty having their voices heard, such as the disadvantaged. It is not a career-enhancing move at a time when many media proprietors have decided that a key to improving profits lies with their so-called AB audiences.

For those not up-to-date with marketing jargon, AB is shorthand for the affluent professionals so beloved by cashed-up advertisers. The theory goes that media outlets that attract audiences at the AB end of the socioeconomic scale are more likely to win advertisers or, even better, to get away with charging them premium rates. A senior manager at one of Australia's major newspaper groups recently explained why his company is focusing on boosting AB readership rather than total circulation [22]. “A good circulation result is one which attracts the readership we need and advertisers want,” said Mark Scott, Editor-in-Chief of Fairfax's metropolitan newspapers, which include the respected broadsheet, *The Sydney Morning Herald*.

“Sure, *The Daily Telegraph* [a tabloid] sells many more copies than *The Sydney Morning Herald*,” said Scott, “but their ad rates are lower because the *SMH [Sydney Morning Herald]* has that AB audience.” Scott said Fairfax's Sunday title *The Sun-Herald* is significantly more profitable at its present circulation level of about 513,000 than it was when it was selling 600,000 copies. “We have held that AB audience so our advertising revenue is up and our costs are lower.”

So what has this to do with how the media report health? Scott explained that his newspapers do extensive market research so they know what the AB market wants to read and how they want it presented. “We create our papers with those readers in mind and shape our marketing and promotions to reinforce their values and interests.” In other words, the allocation of scarce resources in ever more stretched newsrooms is driven by what market researchers tell media managers about what AB audiences want to know about.

This has implications for how the media cover all the areas that affect peoples' health—politics, economics, and education, for example—as well as the coverage of health issues themselves. I haven't seen the market research, but it's not hard to guess what interests AB groups. They might want to know how to stay as healthy, smart, and good-looking as possible for as long as possible. They might want to know which biotech companies are good investments, and might be particularly interested in private health care. They are probably less interested in the needs of indigenous people, prisoners, the homeless, asylum seekers, or the poor, and it's probably a fair bet to say that they are also less interested in the ways in which disadvantaged groups have worse access to health care and prevention efforts.

Some might think this is overly cynical. Perhaps AB people are not all self-centred; perhaps they care about broader issues than those that directly affect their own lives and personal well-being. Nor can the compliance of journalists be assumed. In the chaotic and anarchic world of journalism, there are many who try to do far more with their jobs than to make their bosses wealthy—even if they have to try and “sell” their stories to their news managers on the grounds that the stories will be of interest to the ABs. Many other factors also shape and influence news production. And a truly compelling story is likely to get a run, even if not of direct relevance to the wealthy.

Nonetheless, it is important to remember the commercial imperative when examining media coverage of health. Many initiatives aimed at influencing health coverage target journalists, who are only one component of the media industry. Other powerful forces also shape how health is covered. An analogy can be drawn with efforts to improve the quality and safety of health care, another chaotic industry. Measures aimed at individual clinicians may be helpful in reducing medical errors, but it is also important to look at the broader system in which clinicians work.

## Katherine A. Baverstock: The Media Can Play a Special Role in Providing a Voice for People to Express Their Experiences of Illness

A registered pharmacist for the last 15 years, I was trained in the biomedical model of health, to measure and note signs and symptoms, make assessments, and advise about treatments on the basis of available scientific evidence.

Becoming interested in the portrayal of medicines in the media whilst working in outback Australia (which is grossly underserved by health professionals), I began my doctoral project within this quantitative biomedical tradition. As I found during my literature review, research arising from this tradition assesses media writing about medicines for “quality.” Such research focuses on certain categories of quantitative information about the medicine, such as the indication, associated risks and benefits, outcomes of treatment, contraindications, and cost, that would allow readers to analyse the evidence for themselves and decide whether they should use the medicine.

The research in this area seems to be advocating a position for the health journalist as an educator. Australia's Quality Use of Medicines Strategy [23] has the objective of optimising the use of medicines within the Australian community. It lists the media as a partner in the strategy, together with consumers, health professionals, government, and the pharmaceutical industry. Similar to the other partners, the media have special responsibilities to ensure the quality use of medicines, as described in Box 2. Although many of these responsibilities sit comfortably within the codes of ethics observed by working journalists, some of these responsibilities made me uncomfortable as a health professional. Should journalists be viewed as de facto health educators with the same responsibilities as those of us in the registered health professions?

**Box 2. Australia's National Strategy for Quality Use of Medicines: Responsibilities of the Media**

The media are responsible for the following.

- Ethical and responsible reporting on health-care issues
- Reporting on medicines accurately and attempting to have errors corrected if they occur
- Being aware of the variety of available information sources on medicines and the limitations of each source
- Being aware of the impact of media reports on the use of medicines in the community
- Being aware of issues relevant to the broad context of medicine use, including risks of medicine use, non-drug alternatives, and the cost of medicine use to individuals and society
- Encouraging dissemination of messages that enhance the quality of medication use

Source: [23].

As I progressed in this quantitative framework, I began to feel more and more uncomfortable with the narrow examination of the newspaper stories I had collected for my research. As my analysis continued, it became apparent that the newspaper stories contained themes far richer and more interesting than quantitative information about how drugs work. The stories were an intriguing insight into how the community viewed issues surrounding medicines and the use of medicines. Even more interesting were the narratives about experiences with medicines. I decided to transfer my research to a communications faculty, and explored a far different perspective of medicines in the media. One of my first realisations was that the media are much more than the newspapers, television, and radio focussed on by so many biomedical researchers. They also include new media (the Internet), other print media, and small-scale media, such as leaflets and posters, and even the messages on the pens given out by pharmaceutical representatives.

I would like to propose that rather than act as educators, the media can play a special role in providing a voice for people to express their experiences of illness and their interactions with the technologies of health. The advent of the Internet has democratised the media because this medium is accessible to everyone. The Internet can cross national boundaries and counteract isolation—not only geographic isolation, but also the isolation that may be caused by the experience of chronic illness and not knowing anyone who has lived your experience. People who were unable to have their stories heard within the traditional medical consultation now have a forum where they can be heard and have their stories validated.

There is much research published within the sociological and anthropological literature examining the narrative surrounding health and illness within various types of media. Research now needs to examine how patients use information they find within the media, and whether it does make a difference to the medical encounter. Will an informed and questioning client leave us feeling threatened?

Within the traditional health setting, lengthy communication between medical professionals and clients is often not possible. Many health professionals receive scant training in communication and counselling. The use of media technologies allows our clients to tell their story, a biography that may be ever-changing because of the experience of chronic illness. I would argue, that rather than being much maligned by health professionals, the media should be viewed as a tool that allows healing by facilitating the telling of stories.
