# Correction: What Are the Roles and Responsibilities of the Media in Disseminating Health Information?

**DOI:** 10.1371/journal.pmed.0020321

**Published:** 2005-08-30

**Authors:** Gary Schwitzer, Ganapati Mudur, David Henry, Amanda Wilson, Merrill Goozner, Maria Simbra, Melissa Sweet, Katherine A Baverstock

In *PLoS Medicine*, vol 2, issue 7, DOI: 10.1371/journal.pmed.0020215

The following sentence contains an incorrect number: “Only by massaging the numbers could one figure out that physicians would need to put 700 women on statins to eliminate one cancer case (in medical parlance, this is called number needed to treat).”

The corrected sentence is as follows: “Only by massaging the numbers could one figure out that physicians would need to put 140 women on statins to eliminate one cancer case (in medical parlance, this is called number needed to treat).”

